# Supplementary material for: A workflow of open-source tools for drone-based photogrammetry of marine megafauna
Source: PeerJ. 2025 Aug 12;13:e19768. doi: 10.7717/peerj.19768 (PMC12356183; doi:10.7717/peerj.19768)
Supplement: Supplemental Information 1 [file peerj-13-19768-s001.docx]

*Supplementary Material for*

**A workflow of open-source tools for drone-based photogrammetry of marine megafauna**

K.C. Bierlich,

Joshua Hewitt, Clara N. Bird, David W. Johnston, Julian Dale, Enrico Pirotta, Robert S. Schick, Joshua D. Stewart, Leslie New, Elliott Chimienti, Jeremy Goldbogen, Ari S. Friedlaender, Mauricio Cantor, Leigh G. Torres


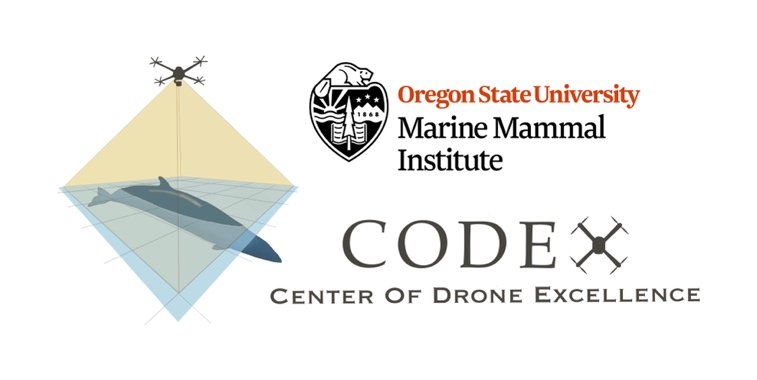


Table of Contents

[1. CODEX Resources 2](#_Toc199364819)

[2. Drone-based Photogrammetry Quick Guide 2](#_Toc199364820)

[3. LidarBoX 2](#_Toc199364821)

[4. Ranking Images for Quality 4](#_Toc199364822)

[5. MorphoMetriX v2 8](#_Toc199364823)

[6. CollatriX v2 11](#_Toc199364824)

[7. DeteX and XtraX 13](#_Toc199364825)

[8. Xcertainty 14](#_Toc199364826)

[9. Multiple Imputation 19](#_Toc199364827)

# 1. CODEX Resources

Marine Mammal Institute’s Center of Drone Excellence (CODEX) at Oregon State University hosts a website that contains the latest version of the photogrammetry workflow, hardware and software tools, tutorials, videos, and available workshops. The CODEX GitHub is where most tools and code will be accessible and updated. The CODEX Listserv connects researchers via email with the updates and releases.

**CODEX website**: <https://mmi.oregonstate.edu/centers-excellence/codex>

**CODEX GitHub**: <https://github.com/MMI-CODEX>

**CODEX Listserv**: <https://lists.oregonstate.edu/mailman/listinfo/codex>

# 2. Drone-based Photogrammetry Quick Guide

We provide a quick guide to help researchers get started on setting up their drone, collecting and processing imagery, and incorporating and propagating measurement uncertainty. We will continue to update the quick guide as new methods and tools are developed and improved. The latest version can be accessed on the CODEX website.

**Quick Guide**:

<https://mmi.oregonstate.edu/centers-excellence/codex/photogrammetry>

# 3. LidarBoX

**Latest updates**: <https://mmi.oregonstate.edu/centers-excellence/codex/software-hardware/lidarbox>

**Design files to build your own**: <https://ir.library.oregonstate.edu/concern/datasets/3n204680g>

**Video tutorial on LidarBoX installation:** <https://oregonstate.app.box.com/folder/183362644982?s=73j7nbjta01s89s85tcy6h3uk19y4hwn>

**Publication**: Bierlich, K., Wengrove, D., Bird, C. N., Davidson, R., Chandler, T., Torres, L. G., & Cantor, M. (2023). LidarBoX: a 3D-printed, open-source altimeter system to improve photogrammetric accuracy for off-the-shelf drones. Drone Systems and Applications. <https://doi.org/10.1139/dsa-2023-0051>

# 4. Ranking Images for Quality

Photogrammetry accuracy can be influenced by image quality. We provide a detailed table, illustrations and example images taken from drone video of gray whales to convey our approach and standards for image quality assessment. We recommend researchers create a similar quality ranking guide specific for their target species. Quality scores follow descriptions from Christiansen et al. (2018) and are adapted for gray whales. Typically, an image with a score of 3 in any attribute category is excluded from the analysis, as well as a score of 2 in at least two of the categories for body arch, body pitch, and body arch (Christiansen et al., 2018). Note that the Bezier curve function in MorphoMetriX photogrammetry software can help measure curved animals, i.e., animals with a quality score > 1.

Table S1. Descriptions of quality attributes and scoring from Christiansen et al., 2018.

| **Attribute** | **Quality Score** | | |
| --- | --- | --- | --- |
|  | **1 (good)** | **2 (medium)** | **3 (poor)** |
| **Camera focus** | Image is clear and in focus with the contour of whale’s body clearly visible | Image is blurry, but clear enough to identify contour of whale’s body | Image is too burry to clearly identify the contour of the whale’s body |
| **Straightness** | The body axis midline crosses the peduncle close to the center | The body axis midline crosses close to or near the peduncle, even if outside of the peduncle | The body axis midline crosses outside and far from the peduncle |
| **Body arch** | The animal is lying flat in the water with no visible arching of the body. | Slight arching of the body (concave or convex) is visible. The head or peduncle is slightly lifted or dropped. | Significant arching of the body (concave or convex). The head or peduncle is significantly lifted or dropped. |
| **Body pitch** | The body axis is flat in the water and not angled vertically | The body axis of the whale is angled slightly in the vertical plane (up or down) | The body axis of the whale is angled significantly in the vertical plane (up or down) |
| **Body roll** | The blowhole of the whale is aligned with the midline body axis | The blowhole of the whale deviates slightly (<1/3 of the eye width) from the midline body axis of the animal | The blowhole of the whale deviates significantly (>1/3 of the eye width) from the midline body axis) |
| **Length measurability** | Both tip of the rostrum and fluke notch are visible | The tip of the rostrum and/or the fluke notch are slightly obscured but can still be identified | The tip of the rostrum and/or the fluke notch is obscured and cannot be confidently identified |
| **Width Measurability** | The body contour of the animal is clearly visible | The body contour of the whale is mostly visible. Some parts along the body are unclear or obscured but can reliably be extrapolated | The body contour of the whale is mostly or totally unclear and obscured parts cannot be confidently extrapolated |


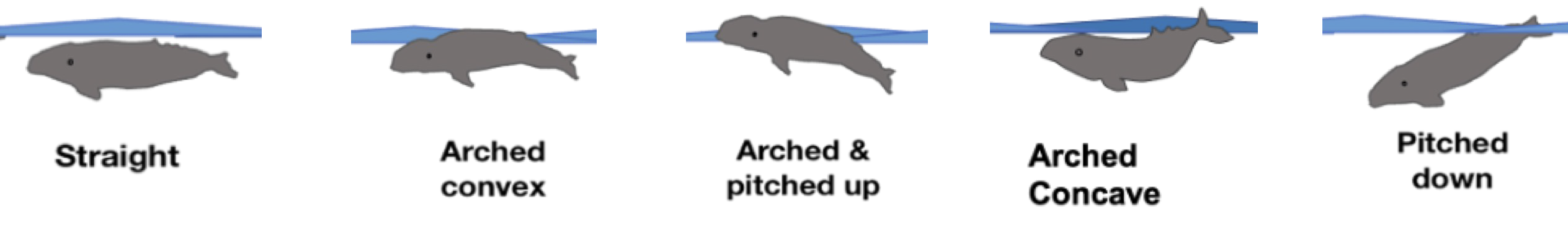


Figure 1S. Side view of different body positions of gray whales commonly seen in drone imagery. *Figure created by Jen-Hsui Ko*.


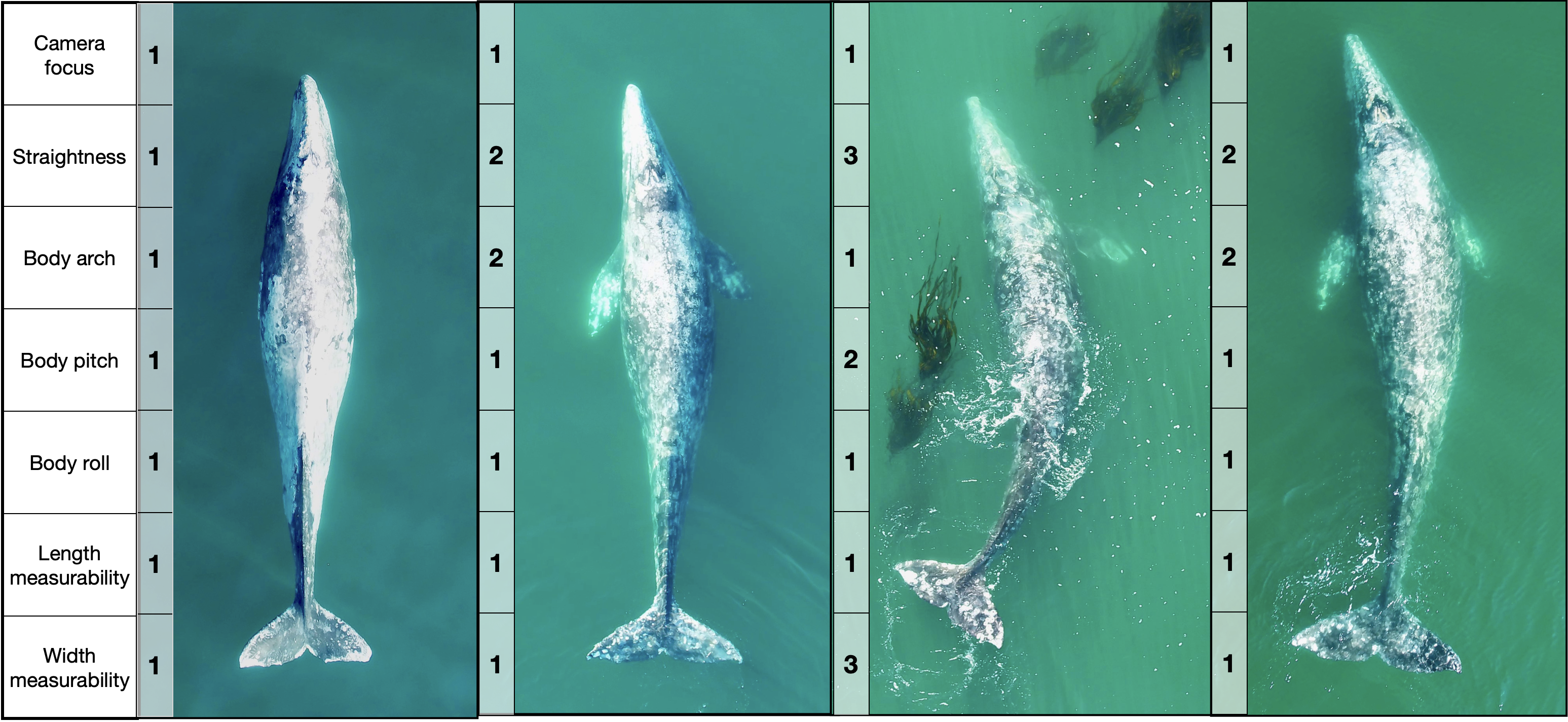


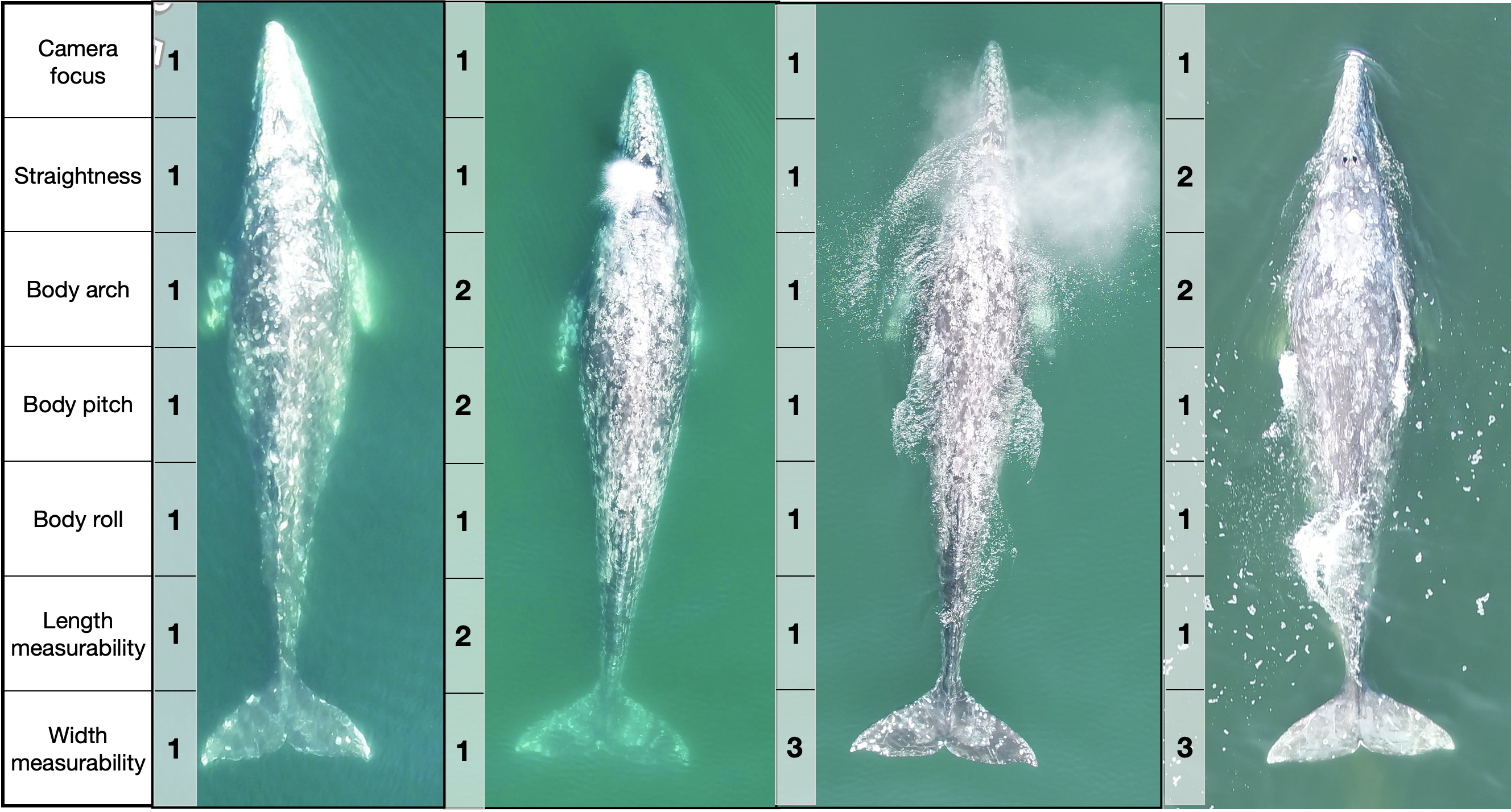


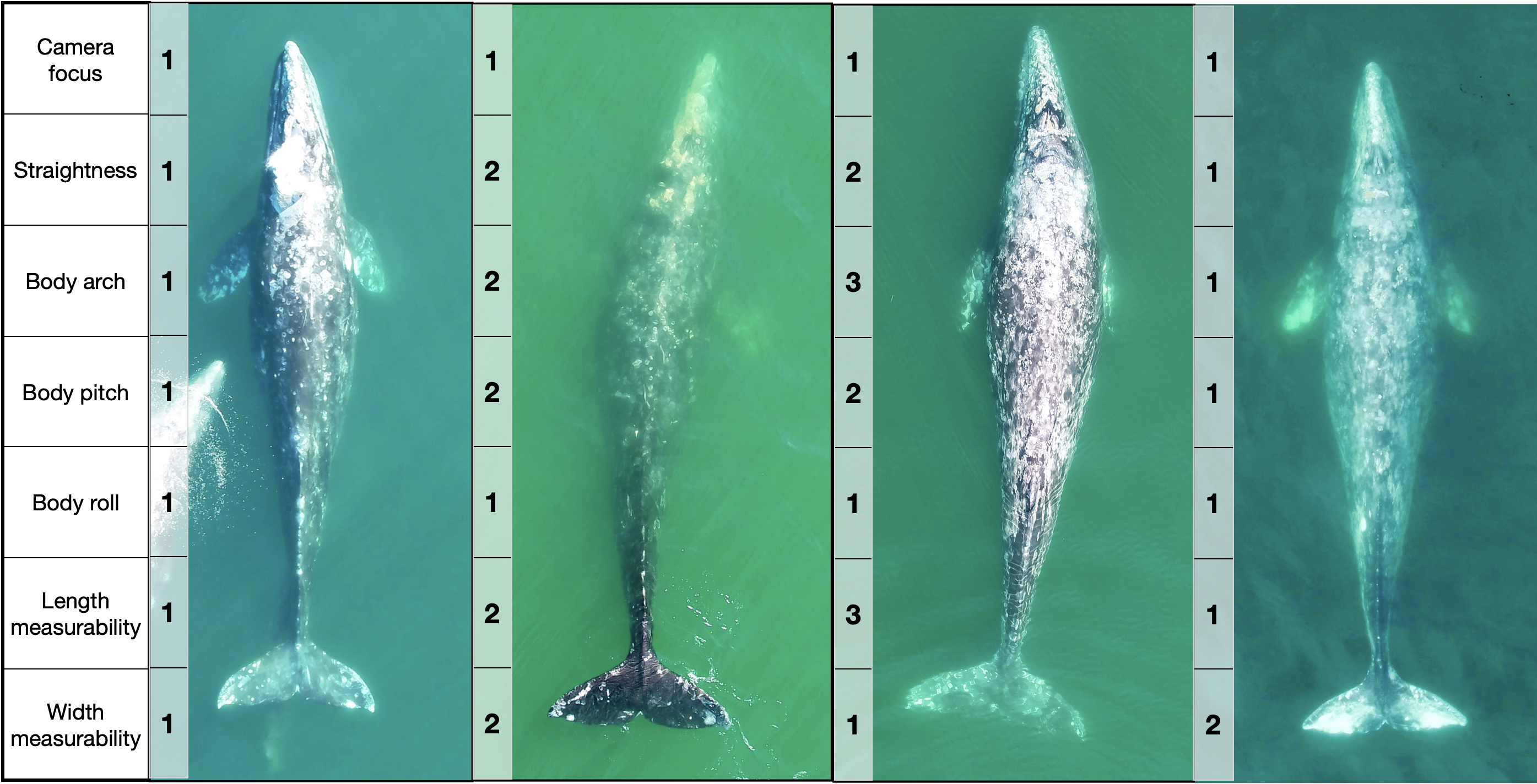


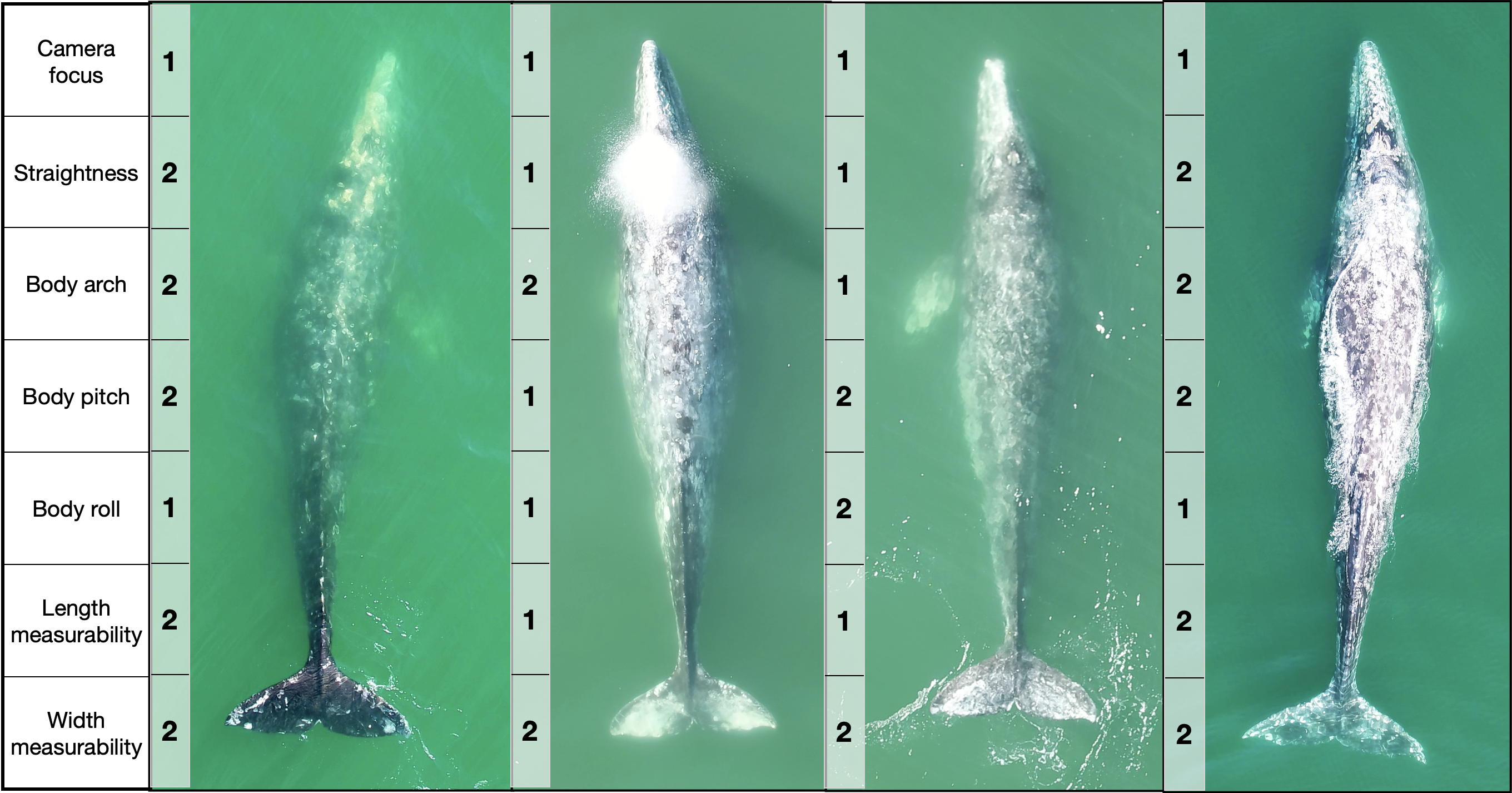


Figure S2. Example images (n = 16) of gray whales with different quality scores for several different attributes: camera focus, straightness, body arch, body pitch, body roll, length measurability, and width measurability. For each attribute, a quality score of 1 = good, 2 = medium, and 3 = poor. See Table S1 for descriptions of quality attributes.

# 5. MorphoMetriX v2

**CODEX website**: <https://mmi.oregonstate.edu/centers-excellence/codex/software-hardware/morphometrix>

**GitHub**: <https://github.com/MMI-CODEX/MorphoMetriX-V2>

**Download latest version here**:

<https://github.com/MMI-CODEX/MorphoMetriX-V2/releases>

- if you have a Mac download the .dmg, if you have a Windows download the .exe
- **Download the manual** *(includes a tutorial)*: <https://github.com/MMI-CODEX/MorphoMetriX-V2/blob/master/MorphoMetriX_v2_manual.pdf>

**Publication**: Torres, W.I., & Bierlich, K.C. (2020). MorphoMetriX: a photogrammetric measurement GUI for morphometric analysis of megafauna. Journal of Open Source Software, 5(45), 1825. <https://doi.org/10.21105/joss.01825>

**More details**

MorphoMetriX v2 can be downloaded as executable (.exe) and Disk iMaGe (.dmg) files for Windows and MacOS, respectively. The MacOS version is functional for Apple M-series chips and Intel chip processors. A link to the detailed manual of MorphoMetriX v2 is also embedded within the GUI and can also be accessed on GitHub (<https://github.com/MMI-CODEX/MorphoMetriX-V2>).

We have improved existing functions and added several new functions to MorphoMetriX v2 (Figure S1). The ‘Measure Widths’ function automatically divides a length measurement into perpendicular widths based on the designated number of width segments specified by the user (Figure 1, Figure S2). MorphoMetriX v2 now automatically places slidable width points on each perpendicular width line, enabling the user to easily adjust and edit width measurements until satisfied with each placement, whereas in v1 users had to manually click each width point with minimal editing ability. The color, size, and shape of the width points can now be customized to help create contrast with the image and ensure the points do not obscure the view of the animal (Figure S2). ‘Mirror Side’ is a new feature that allows users to calculate the body widths of an animal using the edge of the width of one side (A or B) to the center line (Figure S2). This is particularly useful if one of the sides is obstructed, i.e., from glare, refraction, shadows, and greatly increases the number of measurable images available for analysis. The width measurement assumes symmetry between both sides (A and B) and multiplies the selected side by two to get the full width measurement (i.e., width = side A point to center line * 2). The default is “None”, which measures the distance between the edge of the whale from Side A to Side B. The option chosen by the user is reported in the outputted csv. Note, that mirror side option should be used for only measuring non-curved animals.

MorphoMetriX v2 measurement outputs are now exported in both meters and in pixels and saved in long format csv, for a more interpretable output file that can easily be linked with additional metadata. Finally, if MorphoMetriX v2 crashes, a crash report is automatically generated and saved so that it can be uploaded as an “Issue” report on GitHub to help communicate issues and further improve the program. Ultimately, these changes greatly improve the user experience of MorphoMetriX, increase the available sample size, and improve the error reporting functionality, making this update a valuable and significant change.


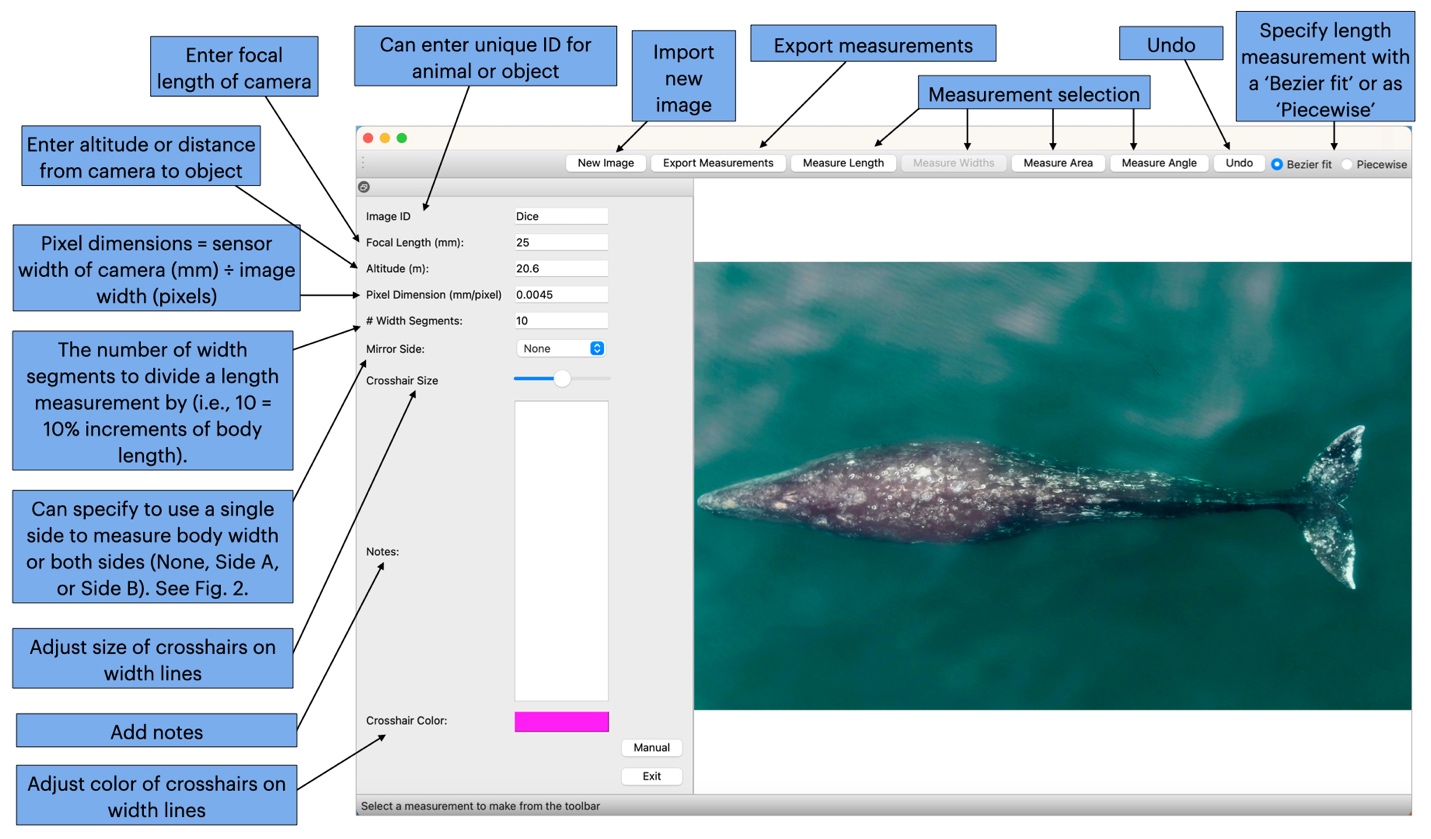


Figure S2. MorphoMetriX v2 with description of functions and inputs in the dialog panel.


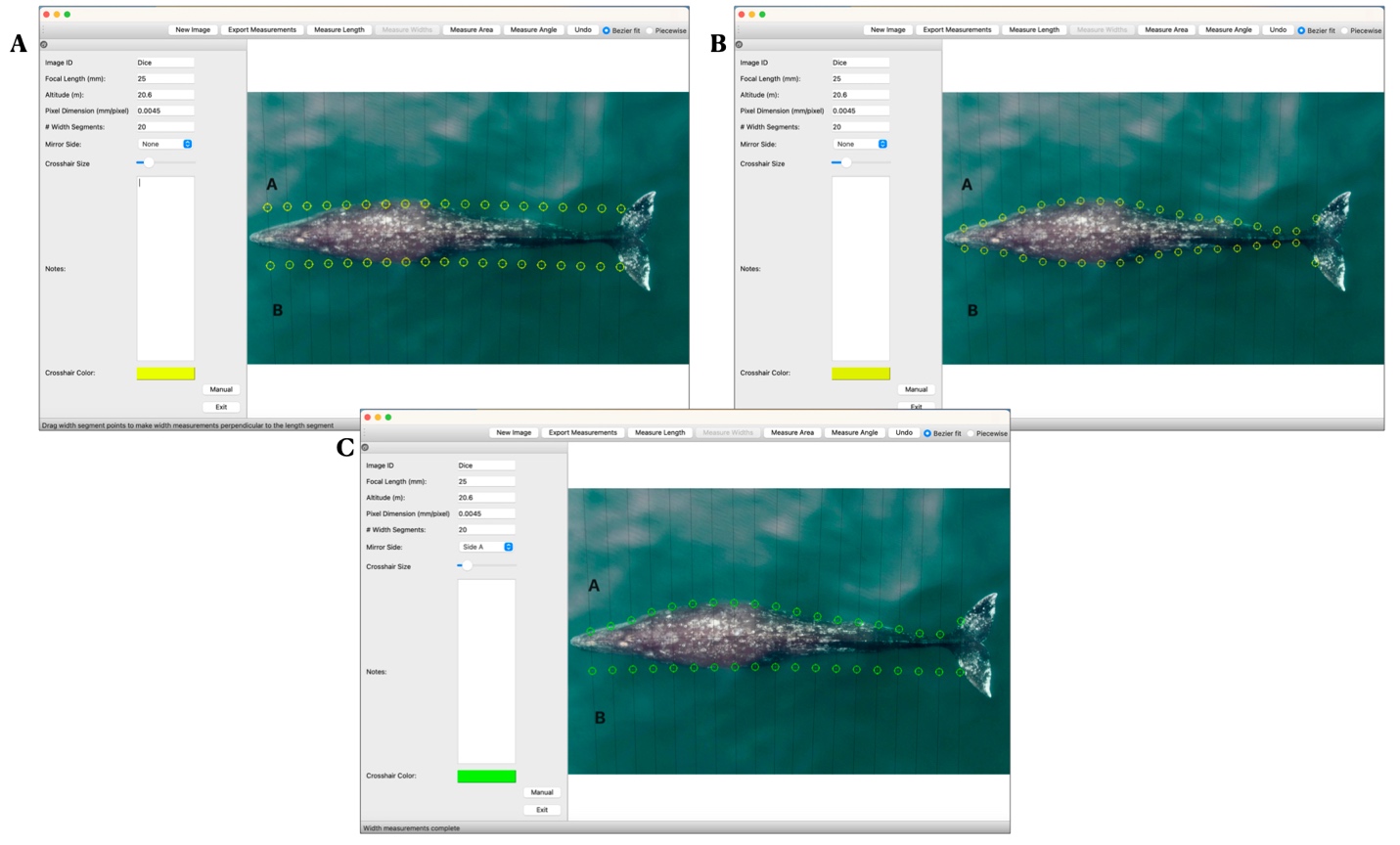


Figure S3. Examples of the enhanced “Measure Widths” function, commonly used to measure body condition of megafauna. A) A total body length measurement is automatically divided into perpendicular widths based on the “# Width Segments” designated by the user (here 20 width segments, or 5% increments of the total body length measurement). Slidable width points are then automatically placed on each perpendicular width. B) The user can then slide and adjust each slidable width point to the desired location, in this example to the edge of the whale’s body on both sides. Here, “Mirror Side” is designated as “None” indicating that each perpendicular width is measured as the distance between side A and B. C) In some cases, one side of the whale’s body may be obstructed, i.e., from glare, refraction, shadows, etc., while the other side is clear and easy to identify. In these circumstances, the user can use “Mirror Side” to select the labeled side of the whale that is clearer to identify (in this example Side A). The total body length measurement is assumed to bisect the animal symmetrically, and so the perpendicular width is then measured as the distance from the width point on the specified side to the intersection of the total body length measurement and multiplied by two.

# 6. CollatriX v2

**CODEX website**: <https://mmi.oregonstate.edu/centers-excellence/codex/software-hardware/collatrix>

**GitHub**: <https://github.com/MMI-CODEX/CollatriX>

**Download latest version here**: <https://github.com/MMI-CODEX/CollatriX/releases>

- **Download the manual** *(includes a tutorial)*: <https://github.com/MMI-CODEX/CollatriX/blob/master/CollatriX_v2_manual.pdf>

**Publication**: Bird, C., & Bierlich, K. C. (2020). CollatriX: A GUI to collate MorphoMetriX outputs. Journal of Open Source Software, 5(51), 2323–2328. <https://doi.org/10.21105/joss.02328>

**More details**

CollatriX v2 can now be downloaded as executable (.exe) and Disk iMaGe (.dmg) files for Windows and MacOS, respectively. The MacOS version is functional for both M1/M2 and Intel chip processors. *Note, IF you are using a Mac, you’ll also need exiftool installed. If you already have it installed, great! If not, download the zipfile [here](https://github.com/cbirdferrer/collatrix-OLD/blob/293d8e37f5a9c82b60726dcb8b4decbad4bb48dc/collatrix/install_exiftool_mac.zip) for an easy download – follow instructions in readme file. More information is available on GitHub (<https://github.com/MMI-CODEX/CollatriX>) where you can find a detailed manual and video tutorial series (linked on the CollatriX ReadMe).

In Collatrix v2 we improved the layout (all tools can be accessed from a home window and each each tool is a single window; Figure S3), added a suite of new tools, and updated the original functions. The new tools are all designed to merge laser altimeter data with drone imagery, including performing the time correction. Furthermore, if the LiDAR value at the time of an image is an NA, the user can tell the merging tools to search for a non-NA altimeter value within a user specified time window. We also updated the original CollatriX function to collate MorphoMetriX v2 outputs and added the elliptical body volume calculation method to the body condition function. Finally, if CollatriX v2 crashes, a crash report is automatically generated and saved to help communicate issues and further improve the program.

​​

Figure S4. Images of the CollatriX v2 (A) home page and (B) body condition function.

**
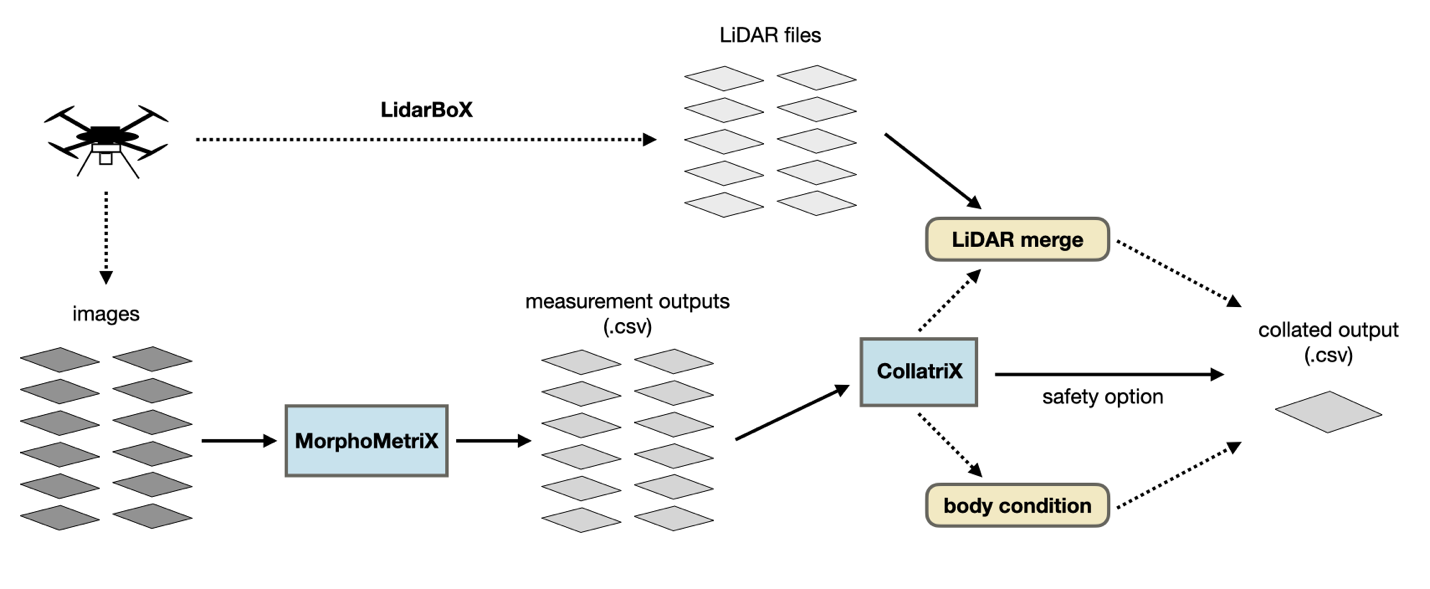
**

Figure S5. CollatriX v2 overview. Graphic depicting how CollatriX version 2 collates MorphoMetriX outputs that can then be merged with LiDAR data and output a final collated file with an option to calculate several common cetacean body condition metrics.

# 7. DeteX and XtraX

Two automated tools to 1) detect and output frames containing whales from drone-based videos (“DeteX”) and 2) extract body length and body condition measurements from input frames (“XtraX”).

**CODEX website**: <https://mmi.oregonstate.edu/centers-excellence/codex/software-hardware/detex-xtrax>

**Video tutorial for setup and running**: <https://media.oregonstate.edu/media/1_01o1wp56>

**Blog providing step-by-step instructions for running**: <https://blogs.oregonstate.edu/gemmlab/2024/05/28/measure-faster-new-tools-for-automatically-obtaining-body-length-and-body-condition-of-whales-from-drone-videos/>

**Publication**: Bierlich, K.C.*, Karki, S.*, Bird, C. N., Fern, A., & Torres, L. G. (2024). Automated body length and body condition measurements of whales from drone videos for rapid assessment of population health. Marine Mammal Science, e13137. <https://doi.org/10.1111/mms.13137> *indicates co-lead author

# 8. Xcertainty

**CODEX website**: <https://mmi.oregonstate.edu/centers-excellence/codex/software-hardware/xcertainty>

**GitHub**: <https://github.com/MMI-CODEX/Xcertainty>

**CRAN**: <https://cran.r-project.org/web/packages/Xcertainty/index.html>

**Installation**

Xcertainty can be installed from CRAN by typing install.packages("Xcertainty") into an R session console. Xcertainty uses the nimble package to run models, which requires a C++ compiler to function properly. The nimble developers provide recommended instructions and some troubleshooting tips at <https://r-nimble.org/html_manual/cha-installing-nimble.html> and <https://github.com/nimble-training/nimble-DFO-2025>, among other places. Alternatively, Windows users can install RTools directly from <https://cran.r-project.org/bin/windows/Rtools/> (be sure to select the installation option box to add RTools to the PATH). Mac users can install Xcode developer tools from the "Additional tools" link at [https://developer.apple.com/xcode/resources/](https://nam04.safelinks.protection.outlook.com/?url=https%3A%2F%2Fdeveloper.apple.com%2Fxcode%2Fresources%2F&data=05%7C02%7Ckevin.bierlich%40oregonstate.edu%7Cd605b2e02aa345fdda3c08dd8735fd0a%7Cce6d05e13c5e4d6287a84c4a2713c113%7C0%7C0%7C638815388609055042%7CUnknown%7CTWFpbGZsb3d8eyJFbXB0eU1hcGkiOnRydWUsIlYiOiIwLjAuMDAwMCIsIlAiOiJXaW4zMiIsIkFOIjoiTWFpbCIsIldUIjoyfQ%3D%3D%7C0%7C%7C%7C&sdata=shLHkLRdmFIse7y7hWPpaggc99G251C3bkVqWlt5c0g%3D&reserved=0), or follow "xcode-select --install" tutorial instructions, like [https://mac.install.guide/commandlinetools/4](https://nam04.safelinks.protection.outlook.com/?url=https%3A%2F%2Fmac.install.guide%2Fcommandlinetools%2F4&data=05%7C02%7Ckevin.bierlich%40oregonstate.edu%7Cd605b2e02aa345fdda3c08dd8735fd0a%7Cce6d05e13c5e4d6287a84c4a2713c113%7C0%7C0%7C638815388609068851%7CUnknown%7CTWFpbGZsb3d8eyJFbXB0eU1hcGkiOnRydWUsIlYiOiIwLjAuMDAwMCIsIlAiOiJXaW4zMiIsIkFOIjoiTWFpbCIsIldUIjoyfQ%3D%3D%7C0%7C%7C%7C&sdata=D0CjH53BPLkx6qgbF77QxhzM39Wn1pgG1ehbCG5HfB4%3D&reserved=0).

**Vignettes**

We provide three vignettes for detailed guidance on using Xcertainty. Below we also provide additional details for Vignette 1 (“Xcertainty” vignette) and using Xcertainty in general.

**Vignette 1 (‘Xcertainty’ vignette)** – basic overview of using Xcertainty. Includes details on using the independent_length_sampler with non-informative priors and the body_condition_function. Also includes several examples on how to view and interpret results. This is the red drone in Figures 3 and 4 of the manuscript. See below for further details. <https://cran.r-project.org/web/packages/Xcertainty/vignettes/Xcertainty.html>

**Vignette 2 (‘Xcertainty informative priors’ vignette)** – detailed example of how and when to use informative priors using the independent_length_sampler. Includes steps for identifying faulty outputs and how to then use the calibration_sampler to help trouble shoot. This is the blue drone in Figures 3 and 4 of the manuscript. <https://cran.r-project.org/web/packages/Xcertainty/vignettes/Xcertainty_informative_priors.html>

**Vignette 3 (‘Xcertainty growth curves’** vignette) – detailed example with code on how to use the growth_curve_sampler, which uses data containing individuals with replicate body length measurements and age information over time. This model fits a Von Bertalanffy-Putter growth curve to observations and incorporates measurement uncertainty associated with multiple drones following Pirotta et al. (2024). Results are shown in Figure 5 of the manuscript. <https://cran.r-project.org/web/packages/Xcertainty/vignettes/Xcertainty_growth_curves.html>

**Additional Details**

**Bayesian model overview:**

Xcertainty builds off the Bayesian statistical framework described in Bierlich et al., (2021a, 2021b) to incorporate total body length and width measurements of individual whales from multiple images.

We first estimate the posterior probability distribution of photogrammetric error parameters (θ) for each drone platform used in data collection using the calibration data of the known-sized objects (*x*) *via*

$$f\left( \theta\right|x)=\frac{f\left( x \right|\theta)f(\theta)}{f(x)}$$

where *f*(*x*|θ) is the likelihood function, *f*(θ) is the prior probability distribution that defines the potential range for θ, *f*(*x*) is the marginal distribution of the measurement data, and *f*(θ|*x*) is the posterior distribution that defines the likely range of θ given data x. We then used the posterior probability distribution for θ as prior information to form a posterior predictive distribution for total body length and width measurements of the whale *via*

$$f\left( x_{new} \right|x)=\int f\left( x_{new} \right|\theta)f\left( \theta| x \right)d\theta$$

where *f*(*x_new_*|θ) is the likelihood function, and *f*(θ|*x*) is the posterior probability distribution estimated from the training data that is set as the new prior probability distribution. The posterior predictive distribution *f*(*x_new_*|*x*) quantifies uncertainty for each measurement (total body length and widths) of the whale, based on the measurement errors from the calibration data. The length and width posterior distributions are then used to calculate a posterior predictive distribution for several different body condition metrics for each individual.

**Xcertainty General steps:**

Xcertainty follows these general steps

1. Prepare calibration and observation data
2. Build sampler
3. Run sampler

Once posterior estimates of body length and widths are obtained, an optional fourth step can be run to estimate several different body condition metrics with uncertainty.

**1. Prepare calibration and observation data**

Once all images of calibration objects and animals have been measured (e.g., using MorphoMetriX v2) and collated (e.g., using Collatrix v2) into a single csv file (one csv for all calibration objects and one for all animal measurements), the Xcertainty function parse_observations will parse wide-format data into a normalized list of dataframe objects that describe the image and measurement data separately (Listing 1). These parsed outputs are then used to build the sampler in the next step. The parse_observations function can be used for both calibration objects and observation (e.g., whales) data, and includes arguments for assigning the appropriate columns in each dataframe that describe the measurements, camera parameters, drone type, and the timepoint of each measurement (if relevant) (Listing 1). Note, input measurements must be in pixels, but if measurements are in meters then the alt_conversion_col argument within parse_observations can be used to assign the altitude column to automatically convert measurements from meters into pixels.


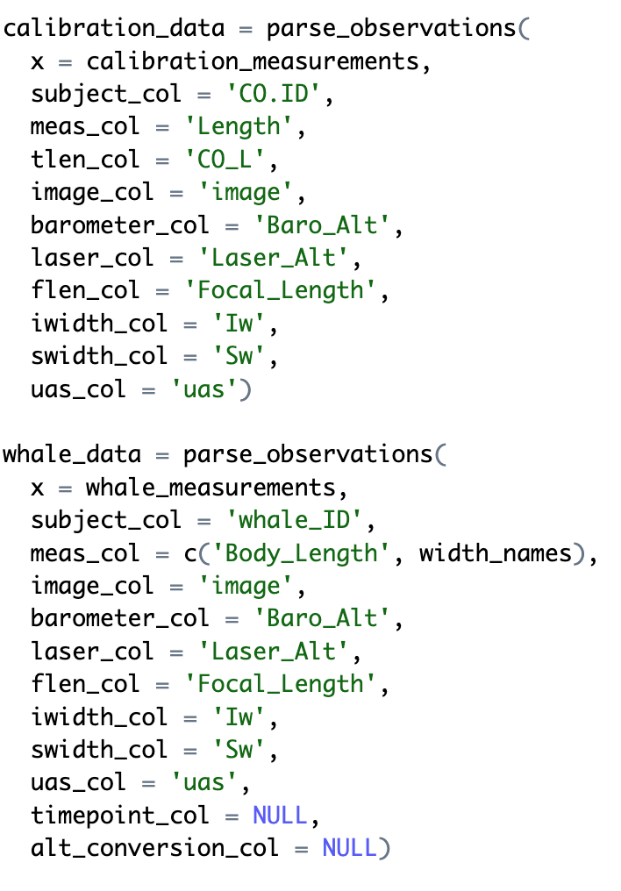


Listing 1. Code used to parse calibration object and whale measurement data using the Xcertainty function parse_observations. Users can input the data (x), the column name(s) of measurements to include (meas_col), images (image_col), barometer altitude (barometer_col), LiDAR/laser altimeter altitude (laser_col), focal length (flen_col), image width (iwidth_col), sensor width (swidth_col), and drone model (uas_col). Note, width_names contains the names of the body width measurements to include.

**2. Build sampler**

Once the calibration object and whale data have been parsed, the next step is to build the model, a Markov chain Monte Carlo (MCMC) sampler. Xcertainty currently includes four built-in samplers:

1. independent_length_sampler, which assumes all Subject/Measurement/Timepoint combinations are independent. This function is well suited for data that contains individuals that either have no replicate samples or have replicate samples that can be treated as independent over time, such as body condition which can increase or decrease.
2. growth_curve_sampler is useful for data that contains replicate samples of individuals with body length measurements and age estimates and is based on the von Bertalanffy-Putter growth model fit in a Bayesian hierarchical framework described by (Pirotta et al., 2024).
3. nondecreasing_length_sampler, uses calibration data to estimate measurements that are assumed to be non-decreasing over time, such as body length across years. This sampler is well suited for individuals with replicate measurements across time points (e.g., years) with no available age information.
4. calibration_sampler only uses calibration data to estimate measurement error parameters, which is useful for checking and comparing variation in training data across different drones and troubleshooting faulty outputs.

For each sampler, combine_observations combines the separately parsed data from the calibration objects and whale measurements (Listing 2).

**2.1 Defining Prior distributions**

Examples 1 and 2 use the independent_length_sampler. Within the independent_length_sampler (as well as the other samplers), there are several arguments to build prior distributions: image_altitude sets the range of altitudes to consider; altimeter_bias sets the range of the potentially consistent offset between the true and observed altitude; altimeter_variance quantifies the consistency between the true and observed altitude (small values indicate typical error tends to be small); altimeter_scaling allows for the possibility that errors in altimeters depend on the true altitude; pixel_variance quantifies the consistency between the true and observed pixel lengths; and object_lengths sets the range of lengths to consider (Listing 2).

We strongly recommend starting with non-informative priors to enable outputs to be driven by the observed data. A non-informative prior distribution for a parameter is an overly wide range or distribution than what is typical or expected. For example, in Listing 2 the range of object_lengths is ‘0.01 to 30 m’, which provides a range far beyond the expected length range of gray whales. Note that the prior distributions in Listing 2 should be appropriate for most datasets, except for increasing the max in object_lengths for larger species, e.g., blue whales. In some cases, using informative priors may be appropriate, especially for datasets with higher error and when the model is overparameterized – see Vignette 2 (the blue drone in Figure 3 of the manuscript).

**3. Run sampler**

Once built, the sampler can be run by setting the number of iterations (niter) and the thinning rate (thin) (Listing 2). This step will produce the posterior distributions for the length and width measurements (Figure 3 in manuscript). Output summaries of the altimeter errors and each individual’s measurements can be viewed and saved, as well as each of the MCMC samples.


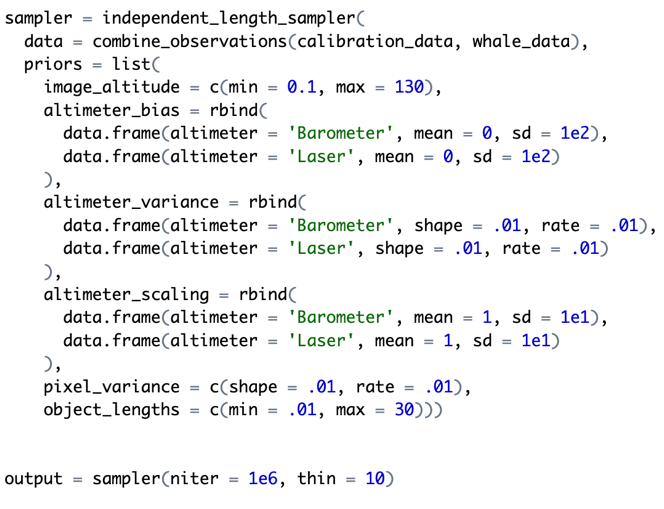


Listing 2. Code used to build and run the sampler in Xcertainty. Here the independent_length_sampler builds a Markov Chain Monte Carlo sampler that is well suited for individuals containing no replicate samples or have replicates that can be treated as independent (e.g., body condition). The prior distribution for each of the parameters listed here are set to be non-informative. The outputs are stored as an object, here as output.

**4. Results**

Results from Example 1 and 2 are illustrated in Figure 3 and 4 of the manuscript. See the vignettes “Xcertainty” and “Xcertainty informative priors” for more details on how to view and interpret results and outputs, as well as how to use the body_condition function, which calculates several body condition metrics.

**Issues**

Users can submit issues encountered while running Xcertainty or suggestions to help improve Xcertainty by submitting a “New issue” on the Xcertainty GitHub page, <https://github.com/MMI-CODEX/Xcertainty/issues>.

**References**

Bierlich, K. C., Hewitt, J., Bird, C. N., Schick, R. S., Friedlaender, A., Torres, L. G., et al. (2021a). Comparing Uncertainty Associated With 1-, 2-, and 3D Aerial Photogrammetry-Based Body Condition Measurements of Baleen Whales. *Front Mar Sci* 8. doi: 10.3389/fmars.2021.749943

Bierlich, K. C., Schick, R. S., Hewitt, J., Dale, J., Goldbogen, J. A., Friedlaender, A. S., et al. (2021b). Bayesian approach for predicting photogrammetric uncertainty in morphometric measurements derived from drones. *Mar Ecol Prog Ser* 673, 193–210. doi: 10.3354/meps13814

# 9. Multiple Imputation

We provide three examples with code that can serve as a template for other researchers to adapt to their own data and analyses. Each example can be found on the CODEX GitHub page: <https://github.com/MMI-CODEX/Examples-Multiple_Imputations>

1. Linear model – morphological relationship between total body length and rostrum to blowhole for Antarctic minke whales (Figure 6 in manuscript).
2. ANOVA – comparing body condition across three blue whale populations
3. Linear model – body condition increases in humpback whales over the Antarctic foraging season across different demographic units.


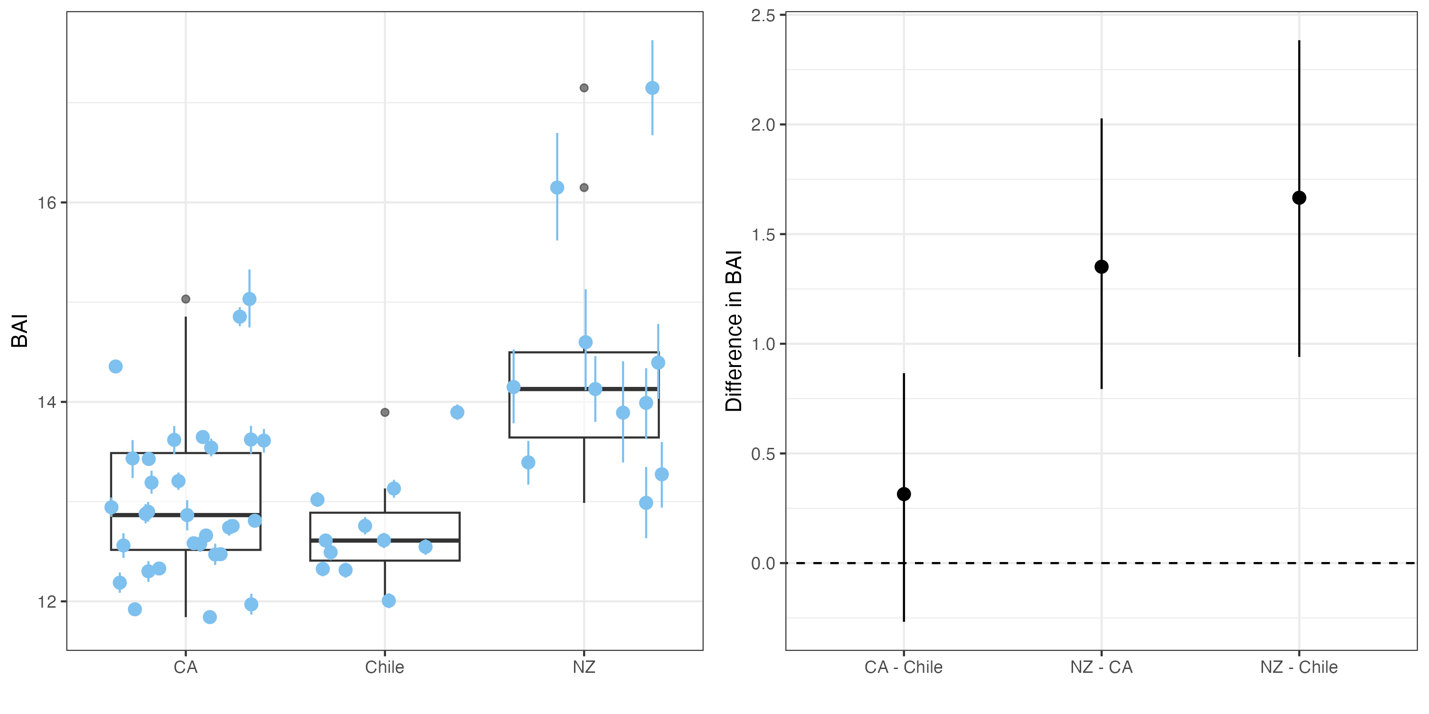


**Figure S6**. Propagating uncertainty in comparing body condition measurements across three blue whale populations from California (CA), Aotearoa New Zealand (NZ), and Chile. A) boxplots of body area index (BAI) with points representing the mean and bars representing the 95% highest posterior density intervals for each individual whale. B) Results from multiple imputation ANOVA and multiple comparisons of BAI across the three populations. Note, an overlap with 0 (dashed line) indicates no significant difference between the two populations. Reproduced from Barlow et al., 2024.


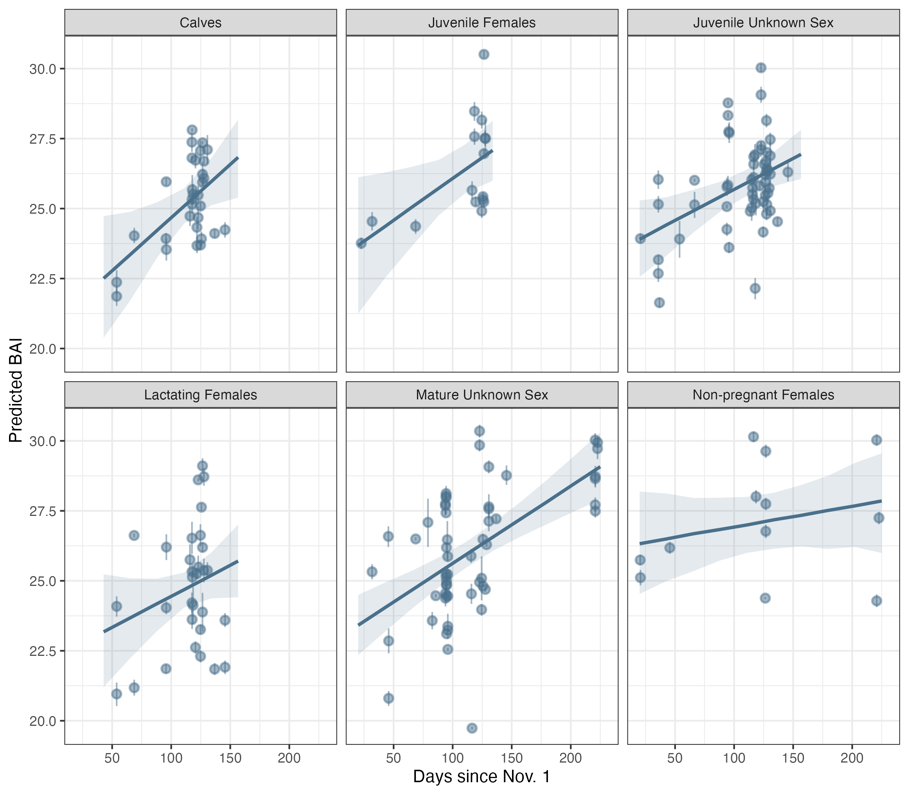


**Figure S7.** Propagating uncertainty in comparing how humpback whale body condition increases over the Antarctic foraging season across different demographic units. Body condition is measured as Body Area Index (BAI) with each point representing the mean and bars representing the 95% highest posterior density intervals for each individual whale. The blue line and shading represent the estimated linear relationship and the 95% credible interval from the multiple imputation linear model. Reproduced from Bierlich et al., (2022).
